# Supplementary material for: Causal relevance of different blood pressure traits on risk of cardiovascular diseases: GWAS and Mendelian randomisation in 100,000 Chinese adults
Source: Nat Commun. 2024 Jul 24;15:6265. doi: 10.1038/s41467-024-50297-x (PMC11269703; doi:10.1038/s41467-024-50297-x)
Supplement: Supplementary file 1 — Supplementary Information [file 41467_2024_50297_MOESM1_ESM.pdf]

Causal relevance of different blood pressure traits on risk of cardiovascular diseases: GWAS and Mendelian randomisation in 100,000 Chinese adults

Members of the China Kadoorie Biobank collaborative group

International Steering Committee: Junshi Chen, Zhengming Chen (PI), Robert Clarke, Rory Collins, Yu Guo, Liming Li (PI), Chen Wang, Jun Lv, Richard Peto, Robin Walters.

International Co-ordinating Centre, Oxford: Daniel Avery, Derrick Bennett, Ruth Boxall, Sue Burgess, Ka Hung Chan, Yiping Chen, Zhengming Chen, Johnathan Clarke; Robert Clarke, Huaidong Du, Ahmed Edris Mohamed, Zammy Fairhurst-Hunter, Hannah Fry, Simon Gilbert, Mike Hill, Pek Kei Im, Andri Iona, Maria Kakkoura, Christiana Kartsonaki, , Kuang Lin, Mohsen Mazidi, Iona Millwood, Sam Morris, Qunhua Nie, Alfred Pozarickij, Paul Ryder, Saredo Said, Sam Sansome, Dan Schmidt, Paul Sherliker, Rajani Sohoni, Becky Stevens, Iain Turnbull, Robin Walters, Lin Wang, Neil Wright, Ling Yang, Xiaoming Yang, Pang Yao.

National Co-ordinating Centre, Beijing: Yu Guo, Xiao Han, Can Hou, Qingmei Xia, Chao Liu, Jun Lv, Pei Pei, Canqing Yu.

10 Regional Co-ordinating Centres: Guangxi Provincial CDC: Naying Chen, Duo Liu, Zhenzhu Tang. Liuzhou CDC: Ningyu Chen, Qilian Jiang, Jian Lan, Mingqiang Li, Yun Liu, Fanwen Meng, Jinhuai Meng, Rong Pan, Yulu Qin, Ping Wang, Sisi Wang, Liuping Wei, Liyuan Zhou. **Gansu** Provincial CDC: Caixia Dong, Pengfei Ge, Xiaolan Ren. Maiji CDC: Zhongxiao Li, Enke Mao, Tao Wang, Hui Zhang, Xi Zhang. **Hainan** Provincial CDC: Jinyan Chen, Ximin Hu, Xiaohuan Wang. Meilan CDC: Zhendong Guo, Huimei Li, Yilei Li, Min Weng, Shukuan Wu. **Heilongjiang** Provincial CDC: Shichun Yan, Mingyuan Zou, Xue Zhou. Nangang CDC: Ziyan Guo, Quan Kang, Yanjie Li, Bo Yu, Qinai Xu. **Henan** Provincial CDC: Liang Chang, Lei Fan, Shixian Feng, Ding Zhang, Gang Zhou. Huixian CDC: Yulian Gao, Tianyou He, Pan He, Chen Hu, Huarong Sun, Xukui Zhang. **Hunan** Provincial CDC: Biyun Chen, Zhongxi Fu, Yuelong Huang, Huilin Liu, Qiaohua Xu, Li Yin. Liuyang CDC: Huajun Long, Xin Xu, Hao Zhang, Libo Zhang. **Jiangsu** Provincial CDC: Jian Su, Ran Tao, Ming Wu, Jie Yang, Jinyi Zhou, Yonglin Zhou. Suzhou CDC: Yihe Hu, Yujie Hua, Jianrong Jin Fang Liu, Jingchao Liu, Yan Lu, Liangcai Ma, Aiyu Tang, Jun Zhang. **Qingdao** Qingdao CDC: Liang Cheng, Ranran Du, Ruqin Gao, Feifei Li, Shanpeng Li, Yongmei Liu, Feng Ning, Zengchang Pang, Xiaohui Sun, Xiaocao Tian, Shaojie Wang, Yaoming Zhai, Hua Zhang, Licang CDC: Wei Hou, Silu Lv, Junzheng Wang. **Sichuan** Provincial CDC: Xiaofang Chen, Xianping Wu, Ningmei Zhang,

Weiwei Zhou. Pengzhou CDC: Xiaofang Chen, Jianguo Li, Jiaqiu Liu, Guojin Luo, Qiang Sun, Xunfu Zhong. **Zhejiang** Provincial CDC: Weiwei Gong, Ruying Hu, Hao Wang,Meng Wan, Min Yu. Tongxiang CDC: Lingli Chen, Qijun Gu, Dongxia Pan， Chunmei Wang, Kaixu Xie, Xiaoyi Zhang.

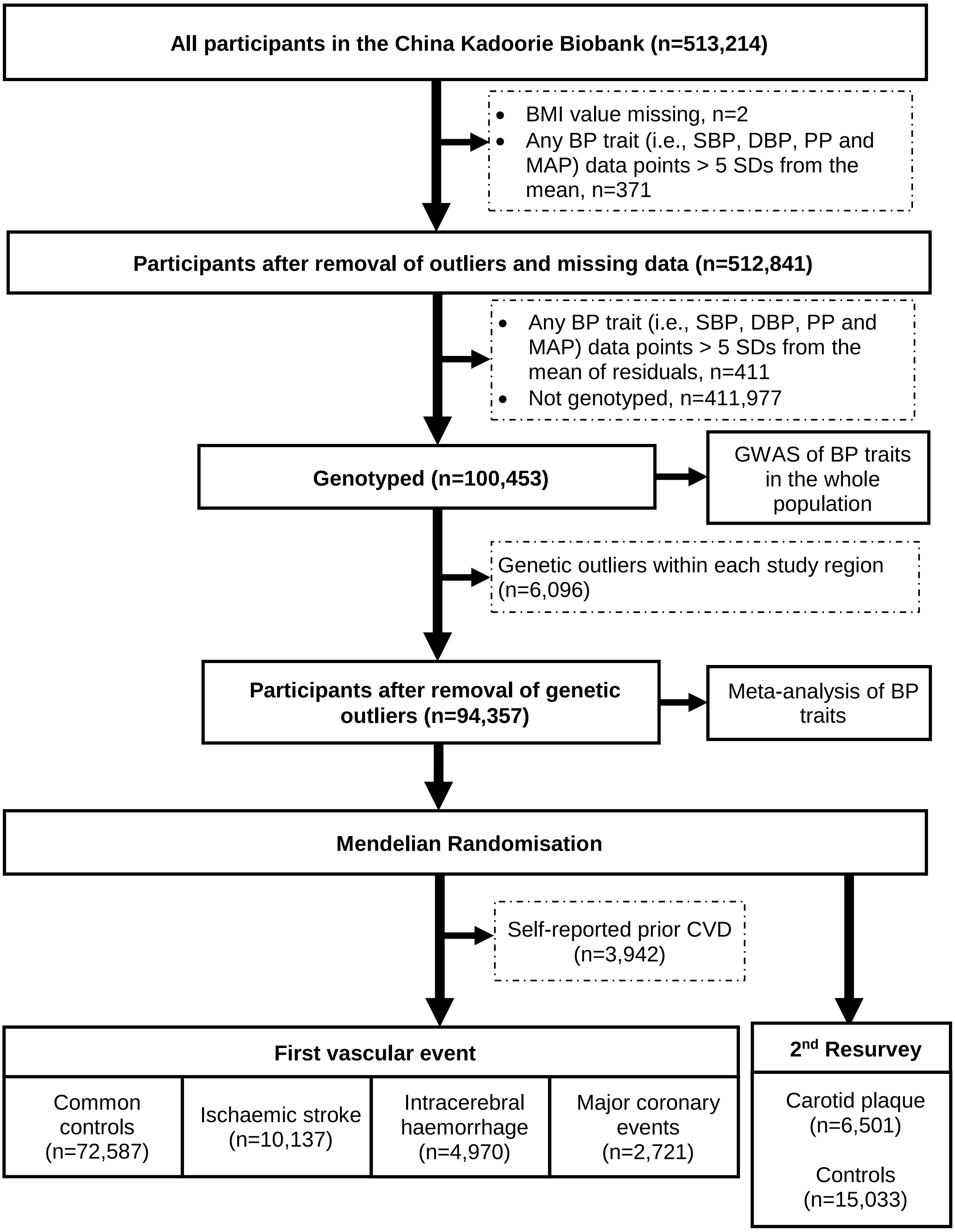

Supplementary Figure 1. Study flow chart for the China Kadoorie Biobank participants. Inclusion/exclusion criteria for the GWAS and Mendelian randomisation analyses are summarised.

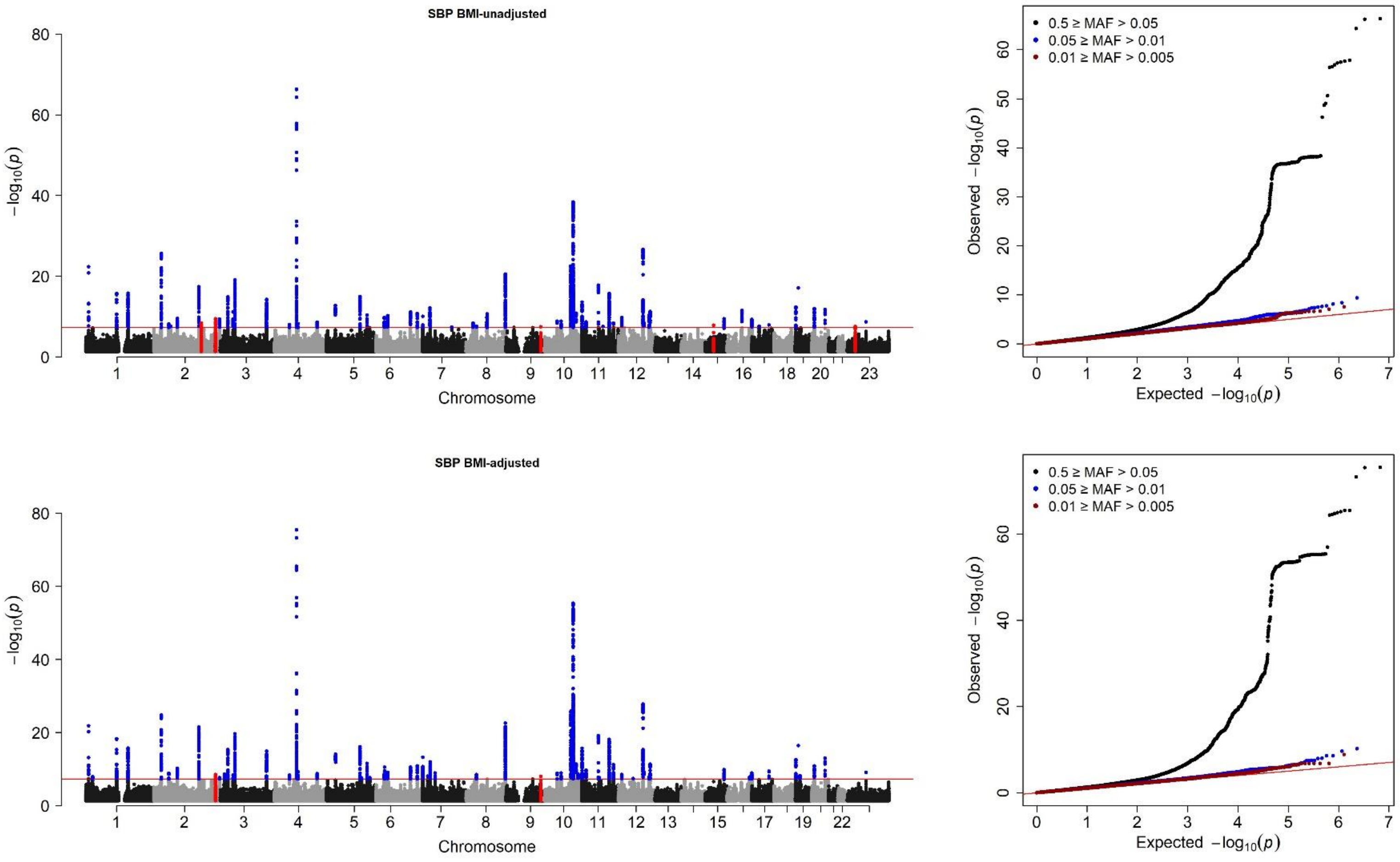

Supplementary Figure 2. Manhattan plots and MAF-stratified quantile-quantile (Q-Q) plots for association with systolic blood pressure. Genome-wide significant loci are highlighted in blue. Novel trait-specific loci are highlighted in red.

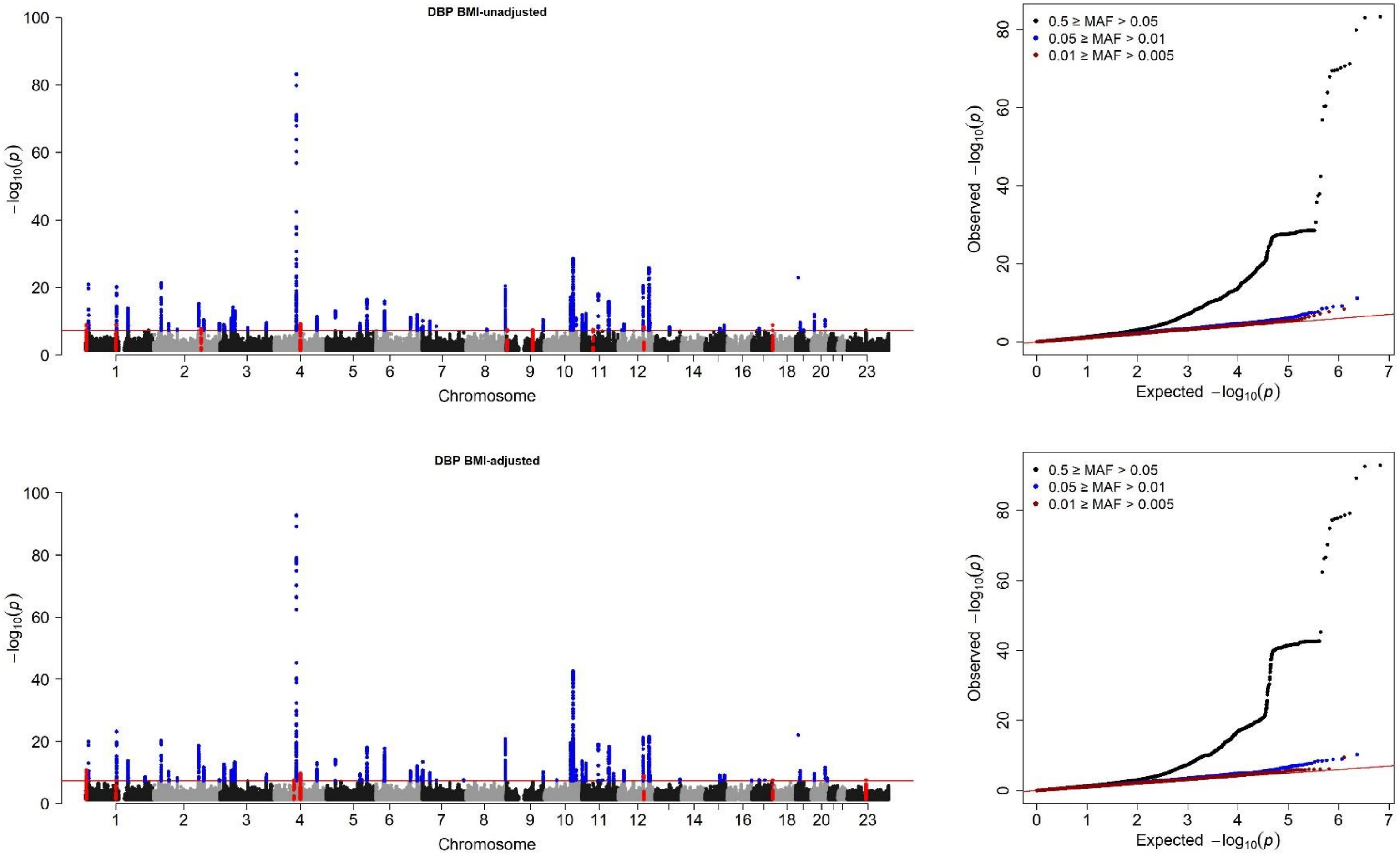

Supplementary Figure 3. Manhattan plots and MAF-stratified quantile-quantile (Q-Q) plots for association with diastolic blood pressure. Genome-wide significant loci are highlighted in blue. Novel trait-specific loci are highlighted in red.

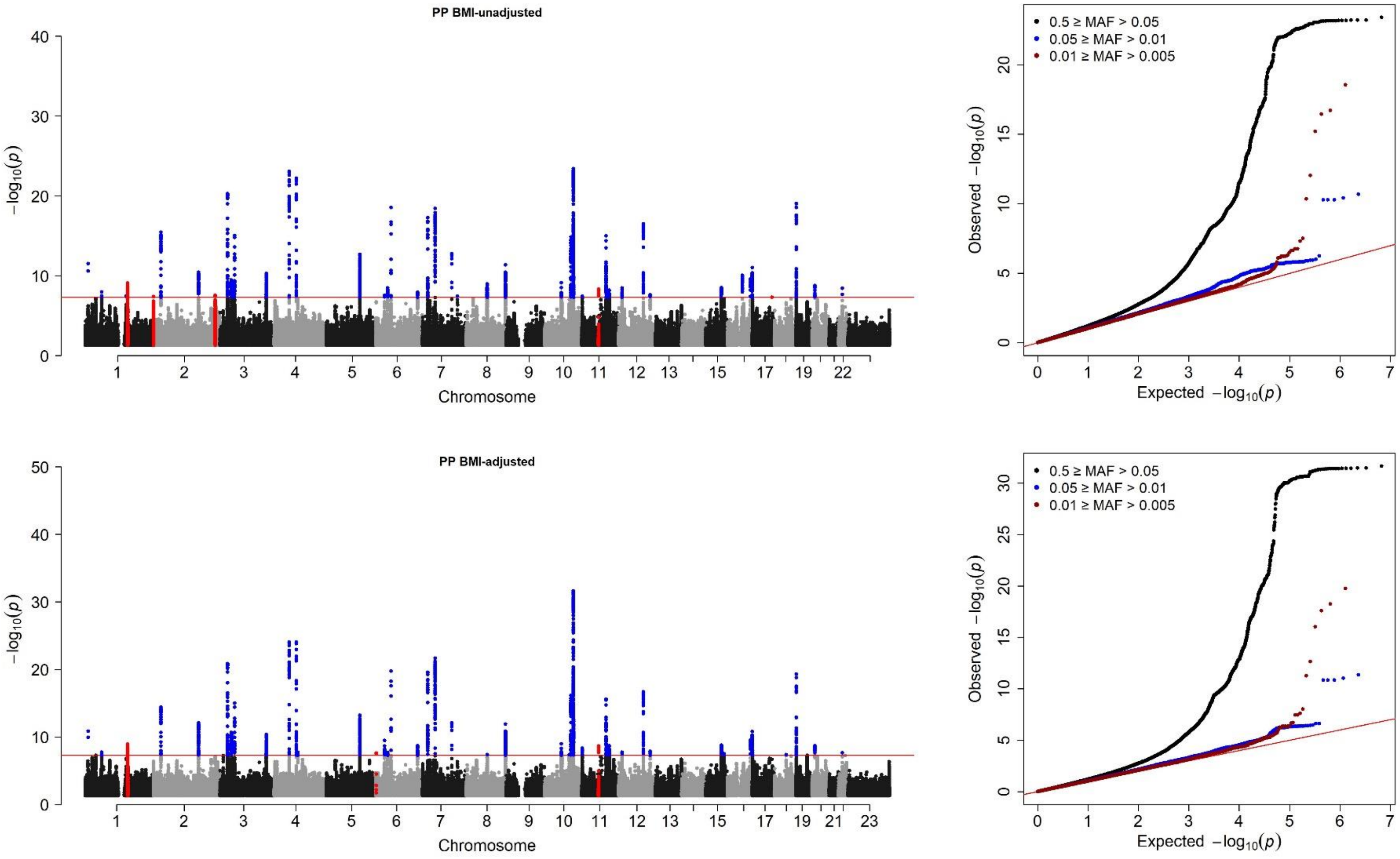

Supplementary Figure 4. Manhattan plots and MAF-stratified quantile-quantile (Q-Q) plots for association with pulse pressure.

Genome-wide significant loci are highlighted in blue. Novel trait-specific loci are highlighted in red.

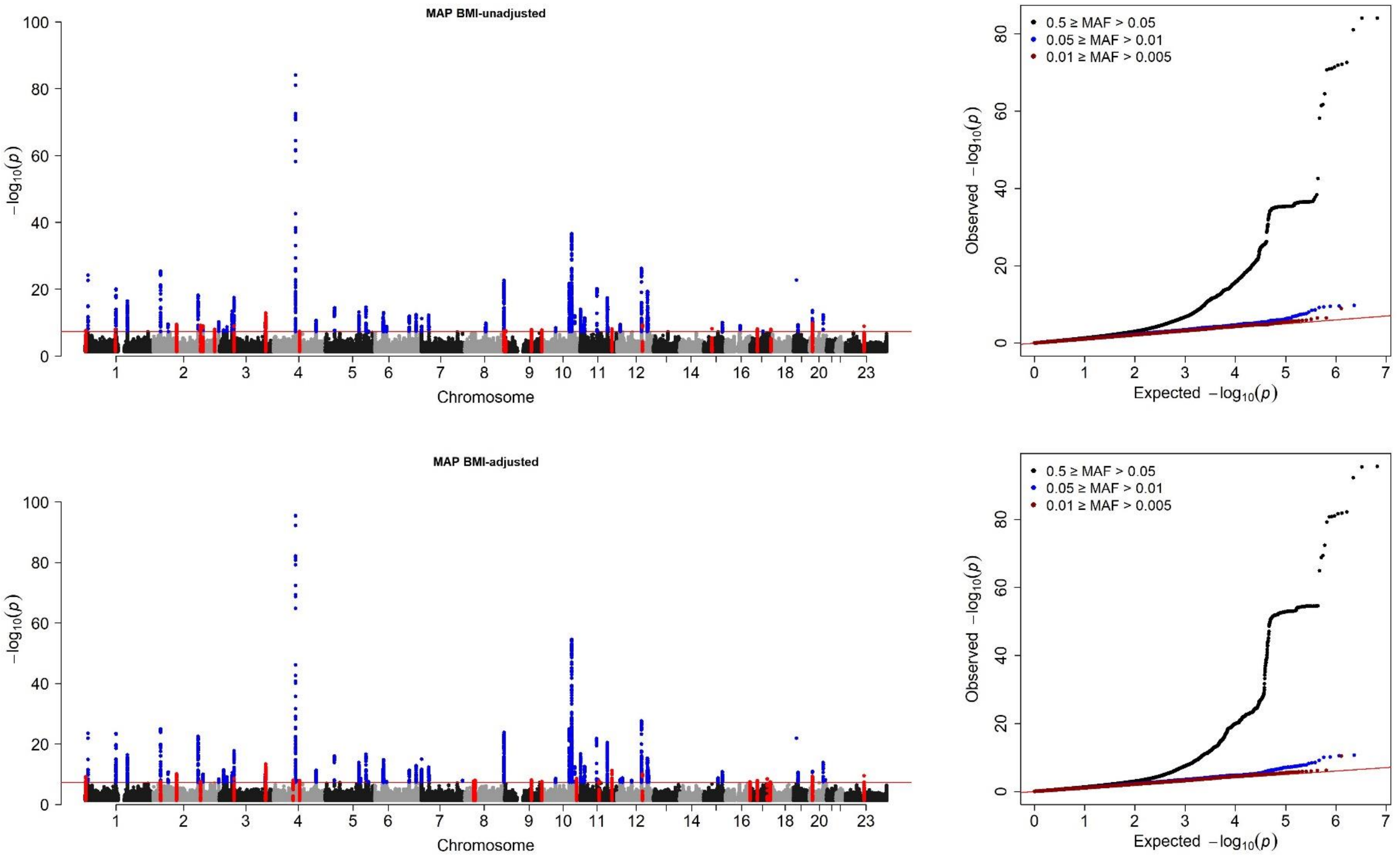

Supplementary Figure 5. Manhattan plots and MAF-stratified quantile-quantile (Q-Q) plots for association with mean arterial pressure. Genome-wide significant trait-specific loci are highlighted in blue. Novel loci are highlighted in red.

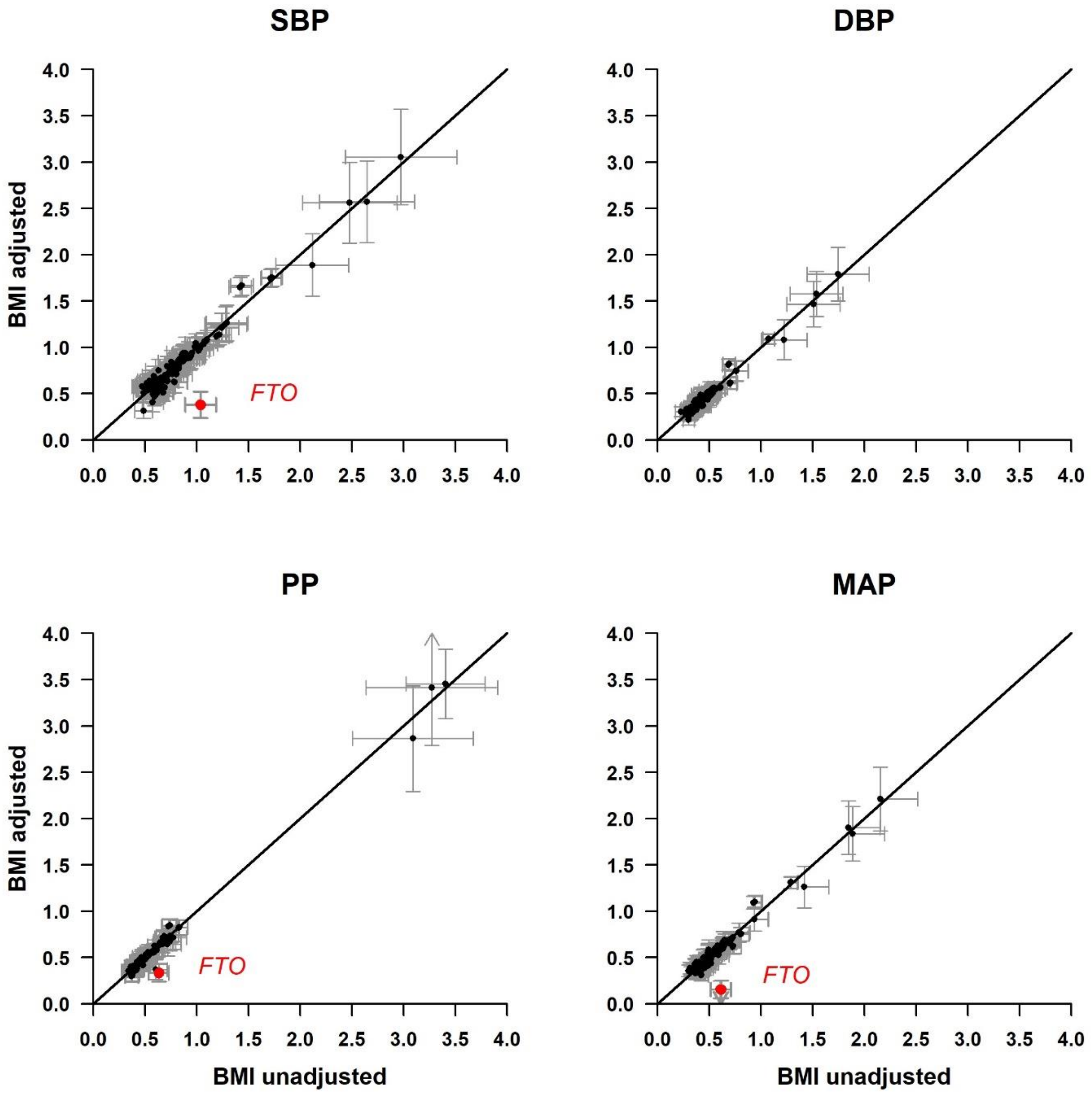

Supplementary Figure 6. Comparison of SNP effect sizes between BMI-adjusted and BMI-unadjusted models. Solid grey lines represent standard errors. SBP indicates systolic blood pressure; DBP, diastolic blood pressure; PP, pulse pressure; MAP, mean arterial pressure; BMI, body mass index.

a

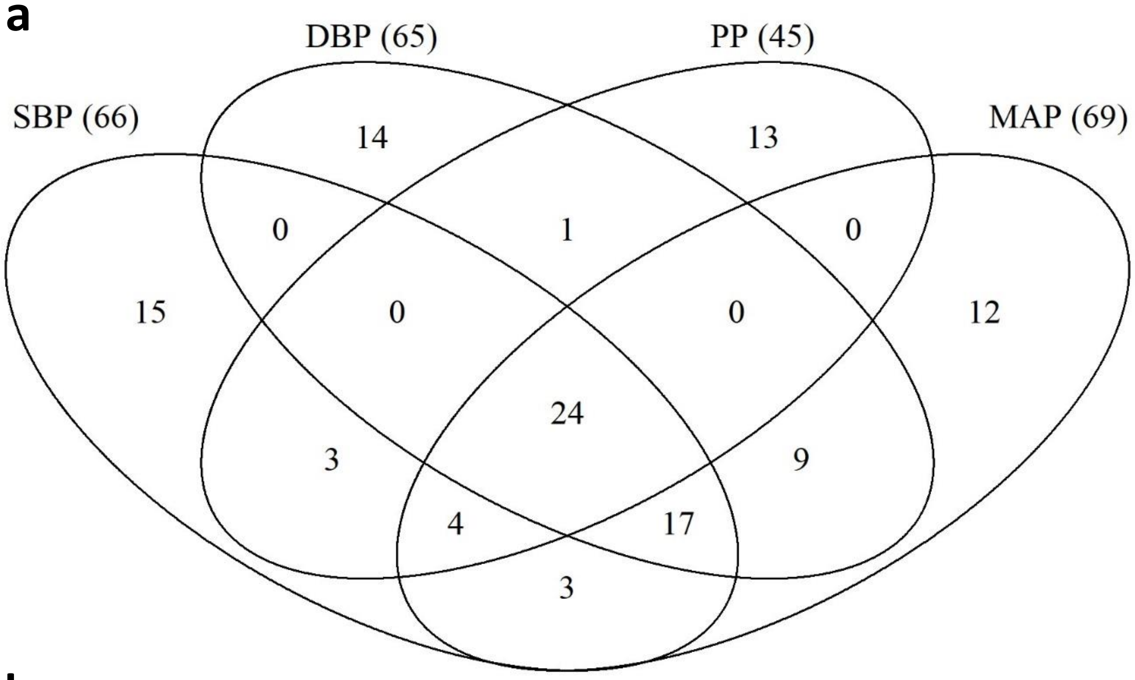

b

Trait	Genetic correlation (LDSC)			
	SBP	DBP	PP	MAP
SBP	1	0.863 (0.013)	0.877 (0.012)	0.961 (0.004)
DBP		1	0.513 (0.040)	0.968 (0.003)
PP			1	0.708 (0.026)
MAP				1

Supplementary Figure 7. Overlap of associations across BMI-unadjusted blood pressure traits. (a) Venn diagram of associations across BMI-adjusted blood pressure traits. The numbers in brackets indicate the total number of loci associated with that BP phenotype. (b) Genetic correlation between 4 traits as assessed by LD score regression.

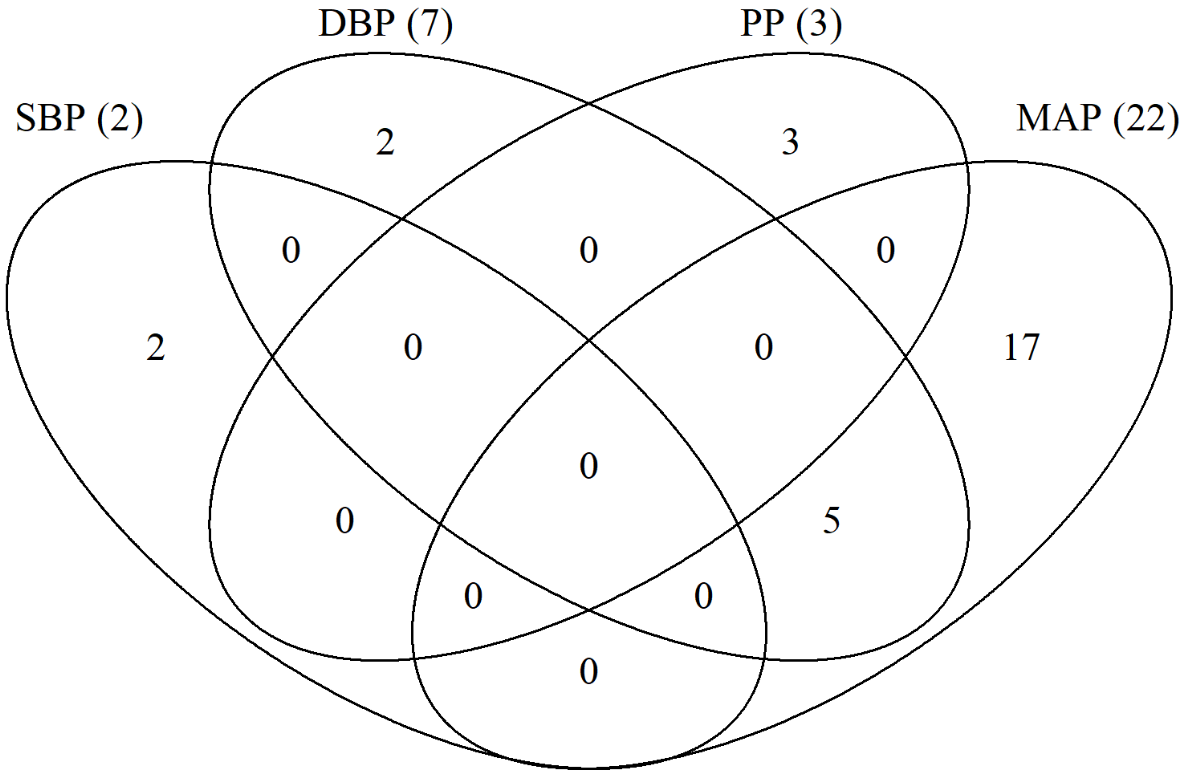

Supplementary Figure 8. Overlap of novel associations across BMI-adjusted blood pressure traits. The numbers in brackets indicate the number of novel loci associated with that BP phenotype. SBP indicates systolic blood pressure; DBP, diastolic blood pressure; PP, pulse pressure; MAP, mean arterial pressure.

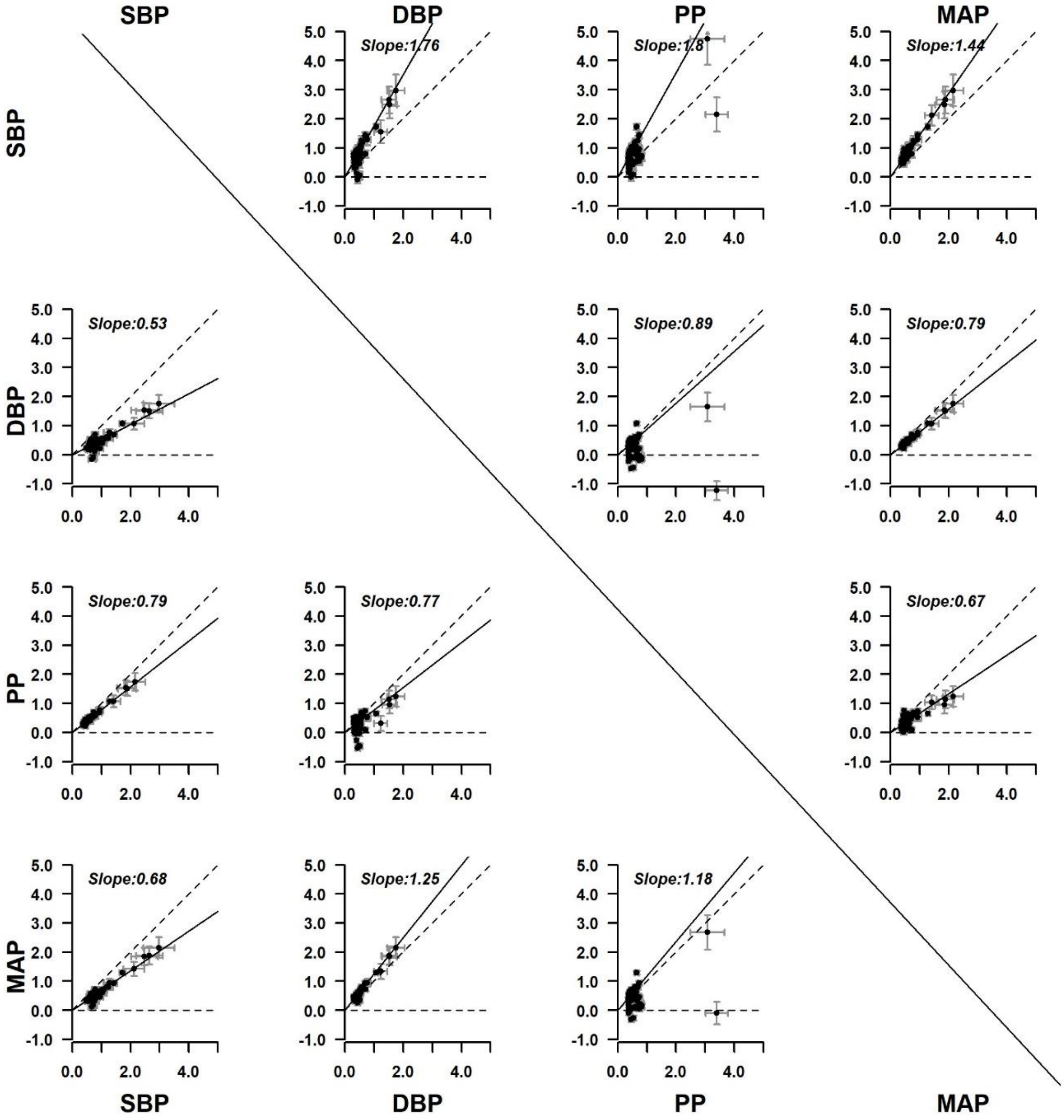

Supplementary Figure 9. Comparison of CKB trait effect sizes for SNPs associated with BMI-adjusted blood pressure traits. Per-allele effect sizes (in mmHg) for SNPs associated with each BP phenotype (in columns) were compared with their effect sizes for the remaining BP phenotypes (in rows). Solid grey lines are standard errors. Dashed diagonal lines represent identity (y = x). Solid black lines are derived from Deming regression, forced through the origin. SBP, systolic blood pressure; DBP, diastolic blood pressure; PP, pulse pressure; MAP, mean arterial pressure

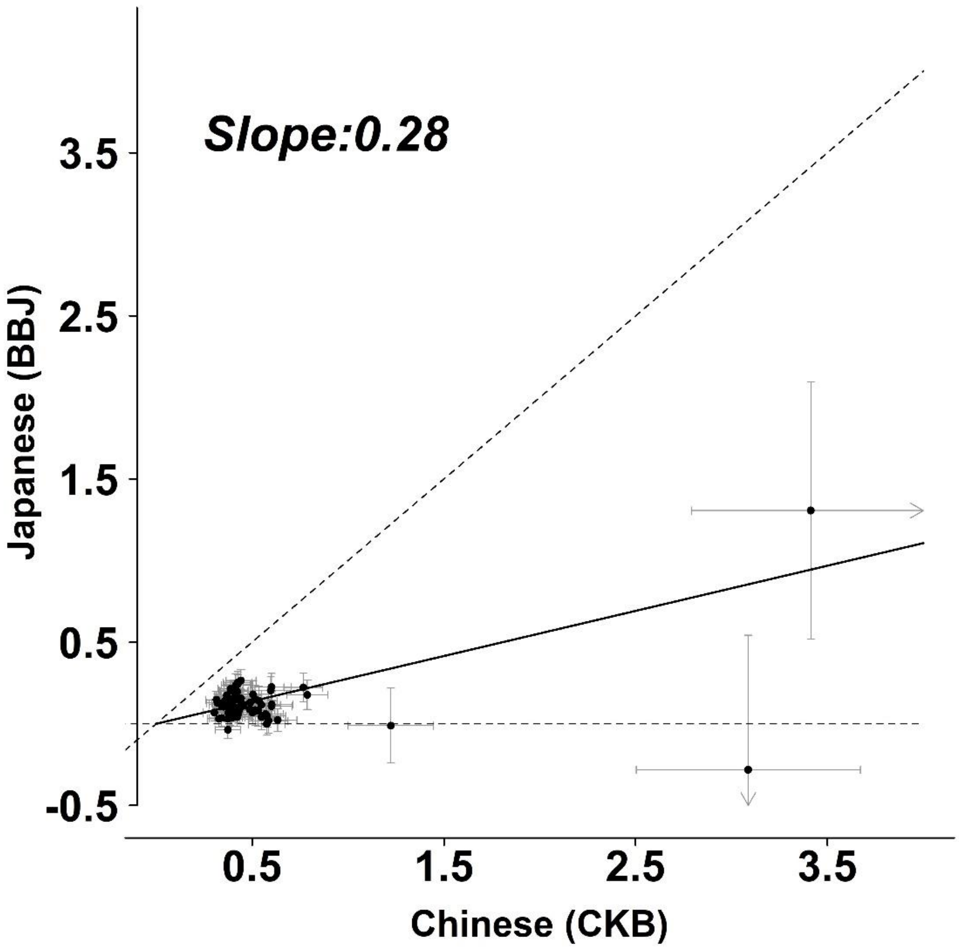

Supplementary Figure 10. Comparison of effect sizes for newly reported blood pressure associations in CKB and BBJ. Per-allele effects are shown in mmHg. Solid grey lines are standard errors. The dashed diagonal line is the identity line (y = x). The solid black line is the Deming regression line forced through the origin.

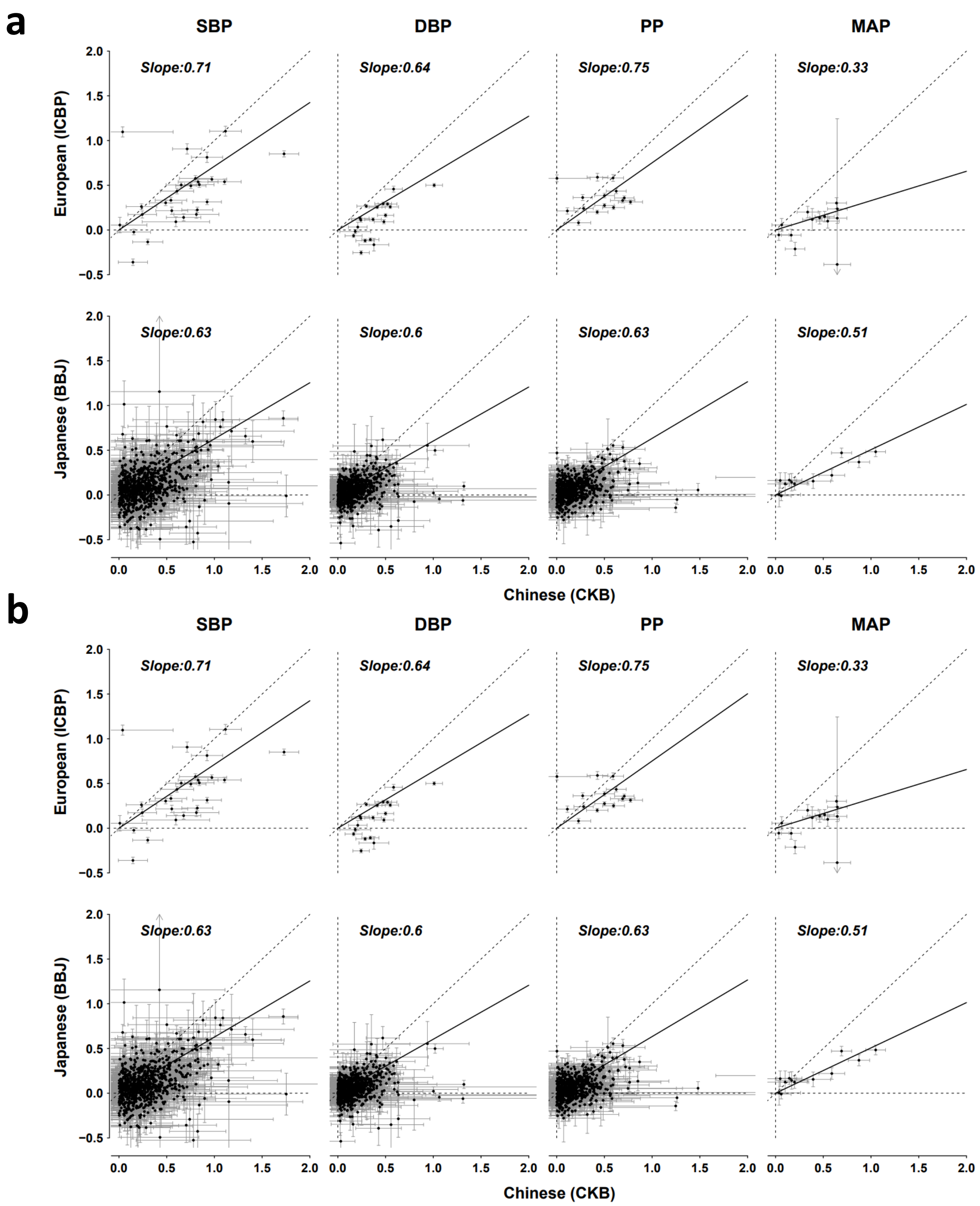

Supplementary Figure 11. Comparisons of variant effect sizes of blood pressure traits between BBJ (BMI-unadjusted), ICBP (BMI-adjusted), and CKB (BMI-unadjusted and BMI-adjusted) cohorts. Comparisons of urban (a) and rural (b) regions of CKB with ICBP and BBJ used variants identified in BBJ and ICBP, respectively. Variant per-allele effects are in mmHg. Solid grey lines are standard errors. The dashed diagonal line is the identity line (y = x). The black solid line is the Deming regression line forced through the origin.

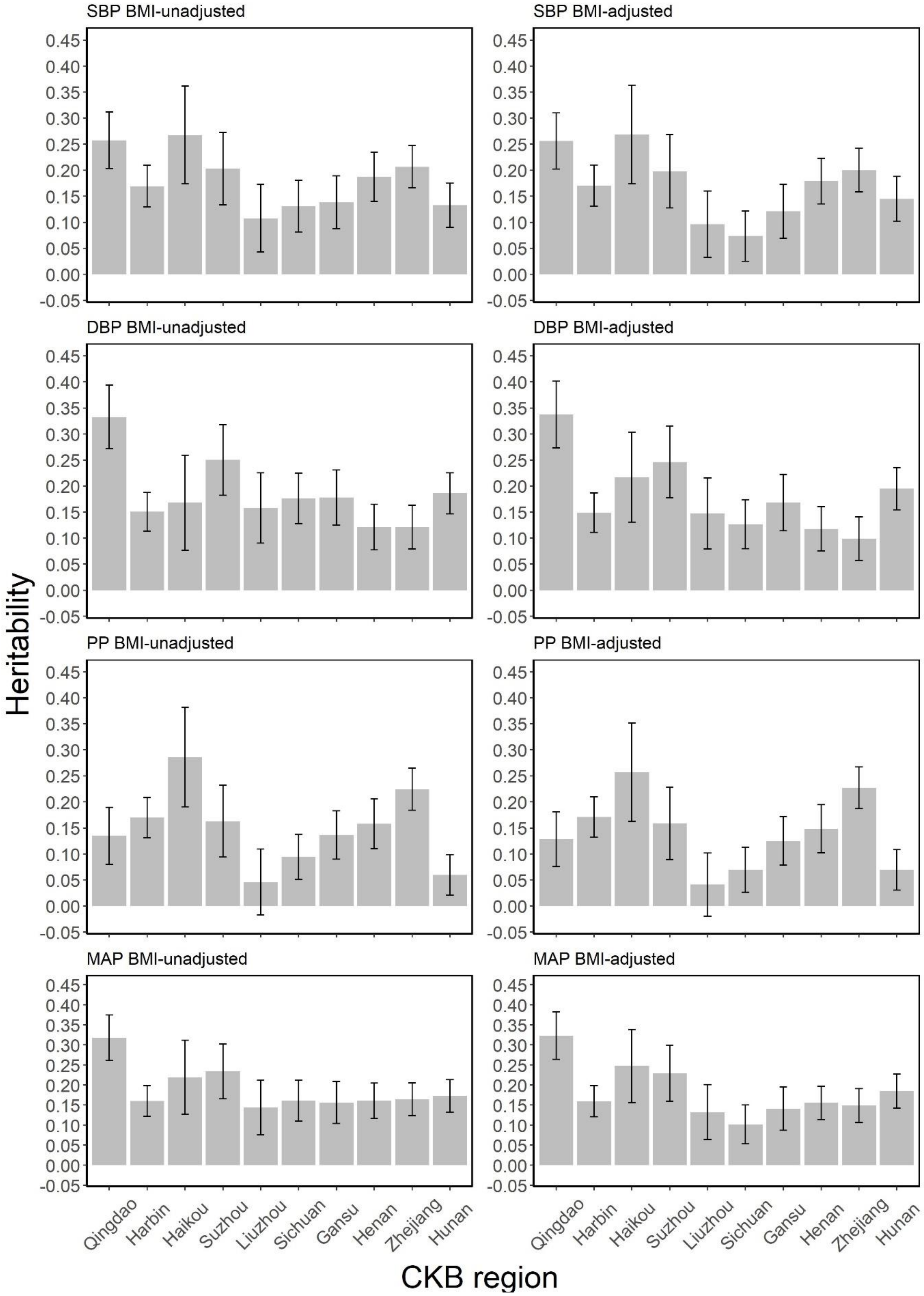

Supplementary Figure 12. Region-specific heritability in CKB. Heritabilities estimated using BOLT-REML. SBP, systolic blood pressure; DBP, diastolic blood pressure; PP, pulse pressure; MAP, mean arterial pressure; BMI, body mass index.

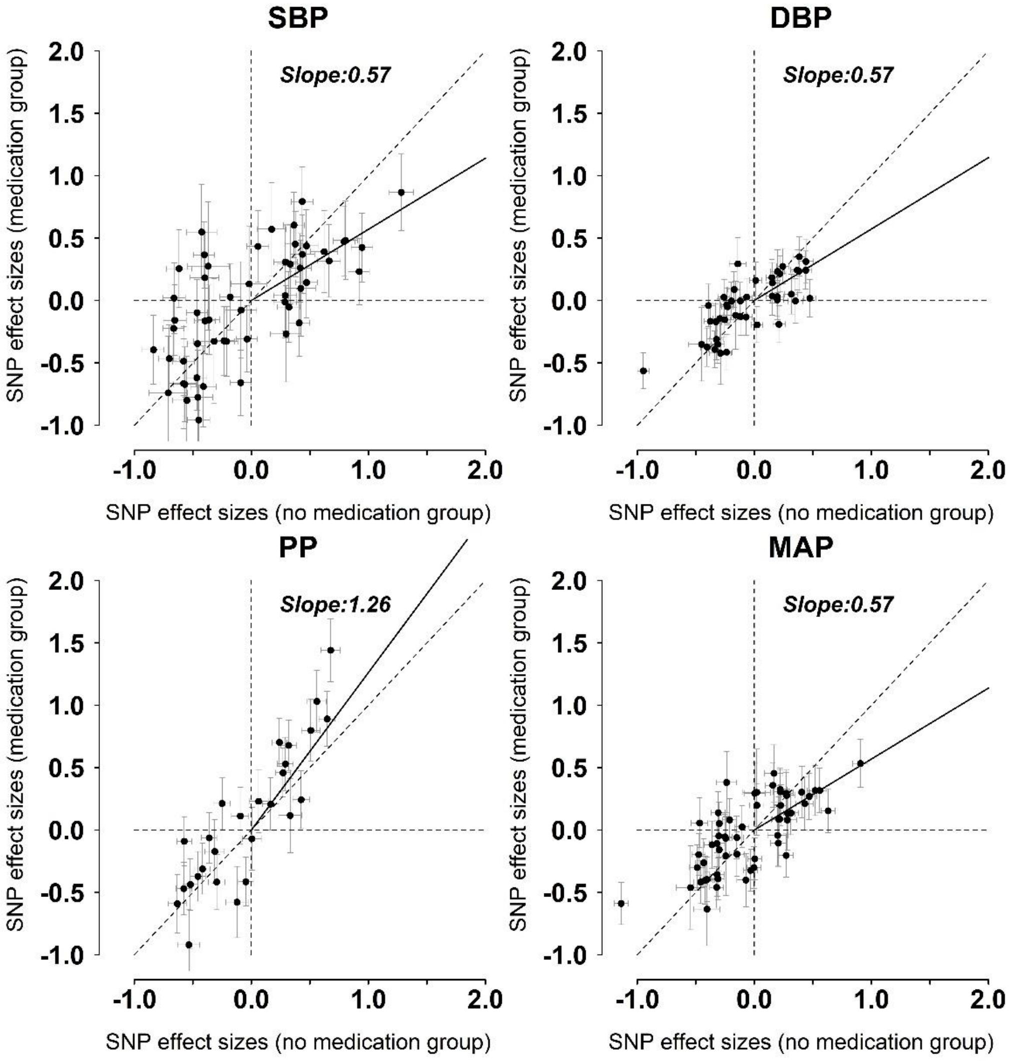

Supplementary Figure 13. Impact of anti-hypertensive medication use on CKB SNP effect sizes. Variant per-allele effects are in mmHg. Solid grey lines are standard errors. The dashed diagonal line is the identity line (y = x). The black solid line is the Deming regression line forced through the origin. SBP, systolic blood pressure; DBP, diastolic blood pressure; PP, pulse pressure; MAP, mean arterial pressure.

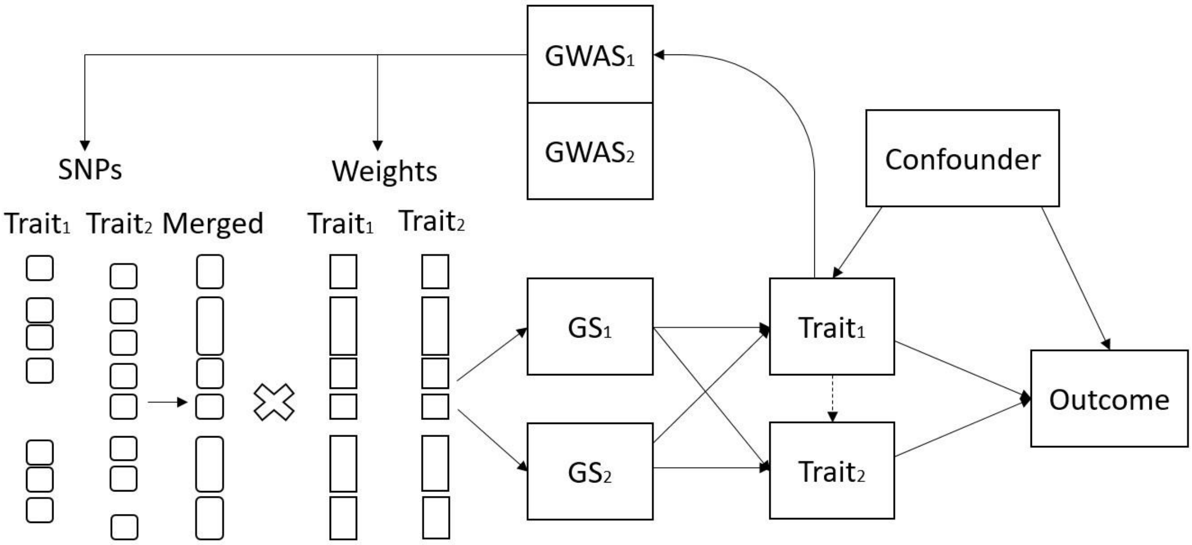

Supplementary Figure 14. Directed acyclic graph demonstrating the principle behind multivariable Mendelian randomization. For each trait, GWAS summary statistics are used to identify associated SNPs and their locus boundaries using LD-based clumping. All loci for the two traits are combined into a single merged list, with overlapping loci merged into larger genomic regions. A single SNP is selected within each region (according to 1 of 3 different methods), and SNP dosages for each trait are weighted by effect sizes estimated in CKB using 100-fold jackknifing. Weighted SNP dosages are summed across genomic regions to create genetic scores (GSs) for each trait. GSs are used to genetically predict Trait 1 and Trait 2, which are then jointly associated with the outcome in a single multivariate model. Any causal effect of trait 1 on trait 2 (dashed arrow) is included in the variant effects on trait 2.

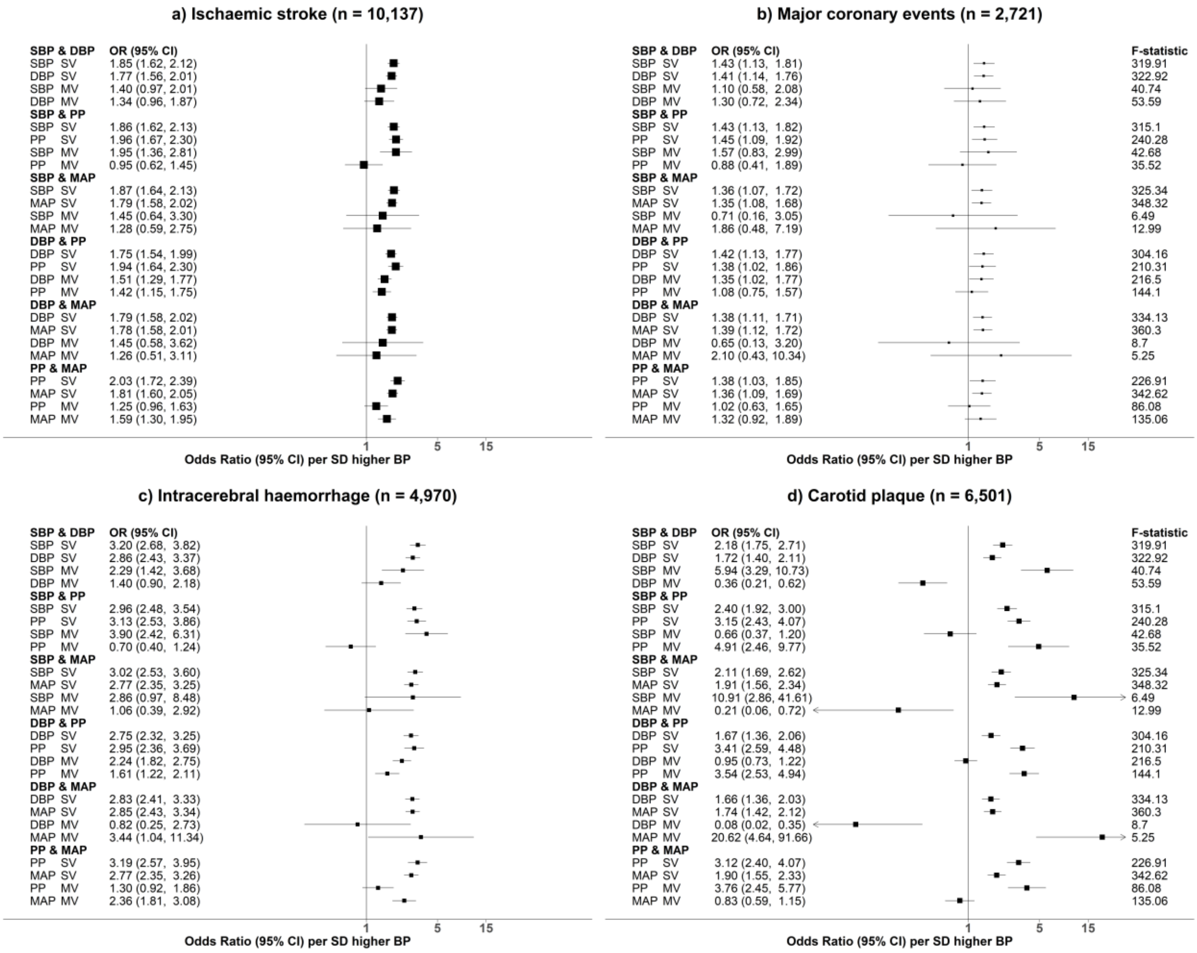

Supplementary Figure 15. Multivariable Mendelian randomisation of blood pressure with major cardiovascular diseases and subclinical atherosclerosis. Effects are shown as odds ratios (95% CI) of disease risk per 1 SD higher BP trait. Loci from two traits were merged and in cases where more than one variant was available in the locus, the variant with the lowest p-value was included as the instrument when constructing the polygenic score. SBP indicates systolic blood pressure; DBP, diastolic blood pressure; PP, pulse pressure; MAP, mean arterial pressure; SV, single variable MR; MV, multi-variable MR. F-statistic was calculated as the average across CKB regions. F-statistic for MV indicates conditional F-statistic.

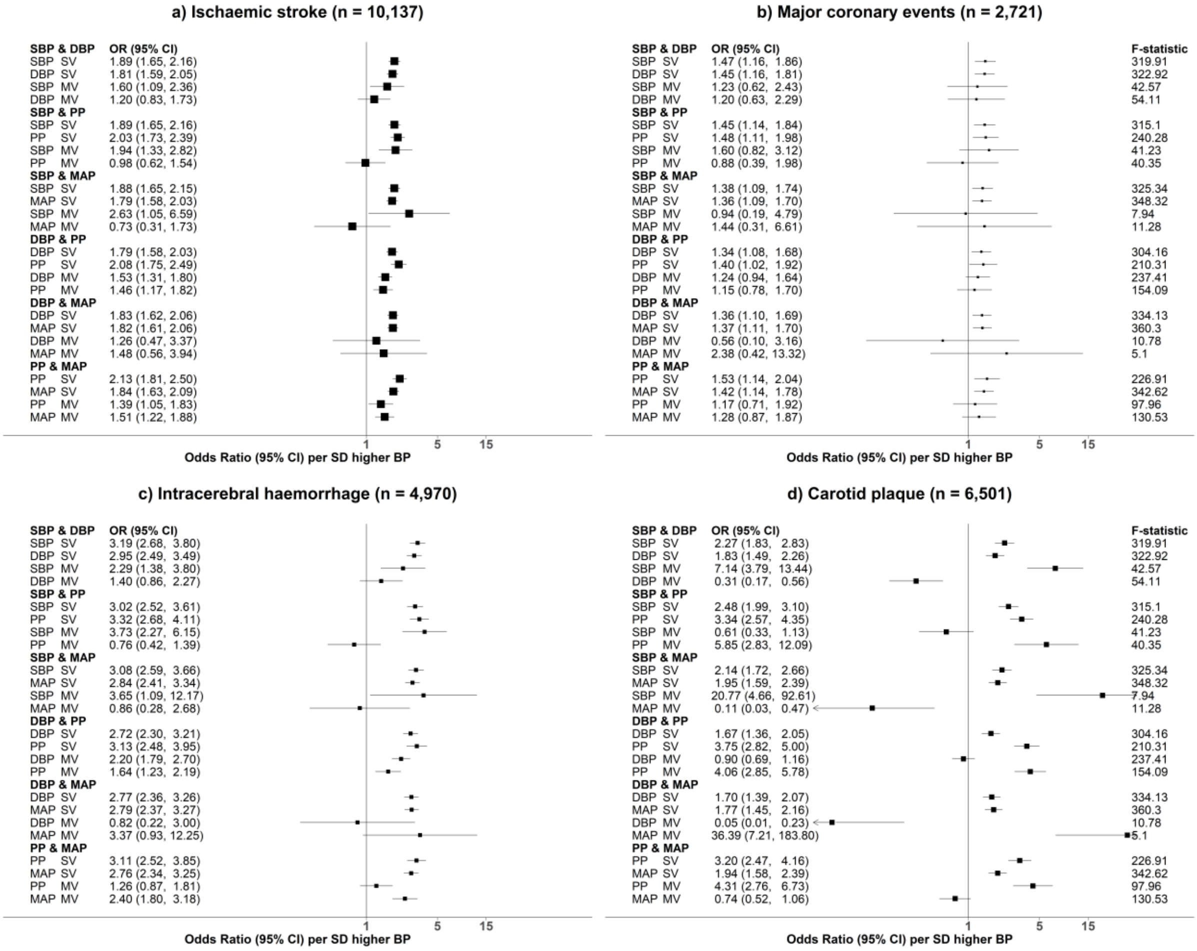

Supplementary Figure 16. Multivariable Mendelian randomisation of blood pressure with major cardiovascular diseases and subclinical atherosclerosis, sensitivity analysis. Effects are shown as odds ratios (95% CI) of disease risk per 1 SD higher BP trait. Loci from two traits were merged and in cases where more than one variant was available in the locus, the variant from the first trait was always included as the instrument when constructing the polygenic score. SBP indicates systolic blood pressure; DBP, diastolic blood pressure; PP, pulse pressure; MAP, mean arterial pressure; SV, single variable MR; MV, multi-variable MR. F-statistic was calculated as the average across CKB regions. F-statistic for MV indicates conditional F-statistic.

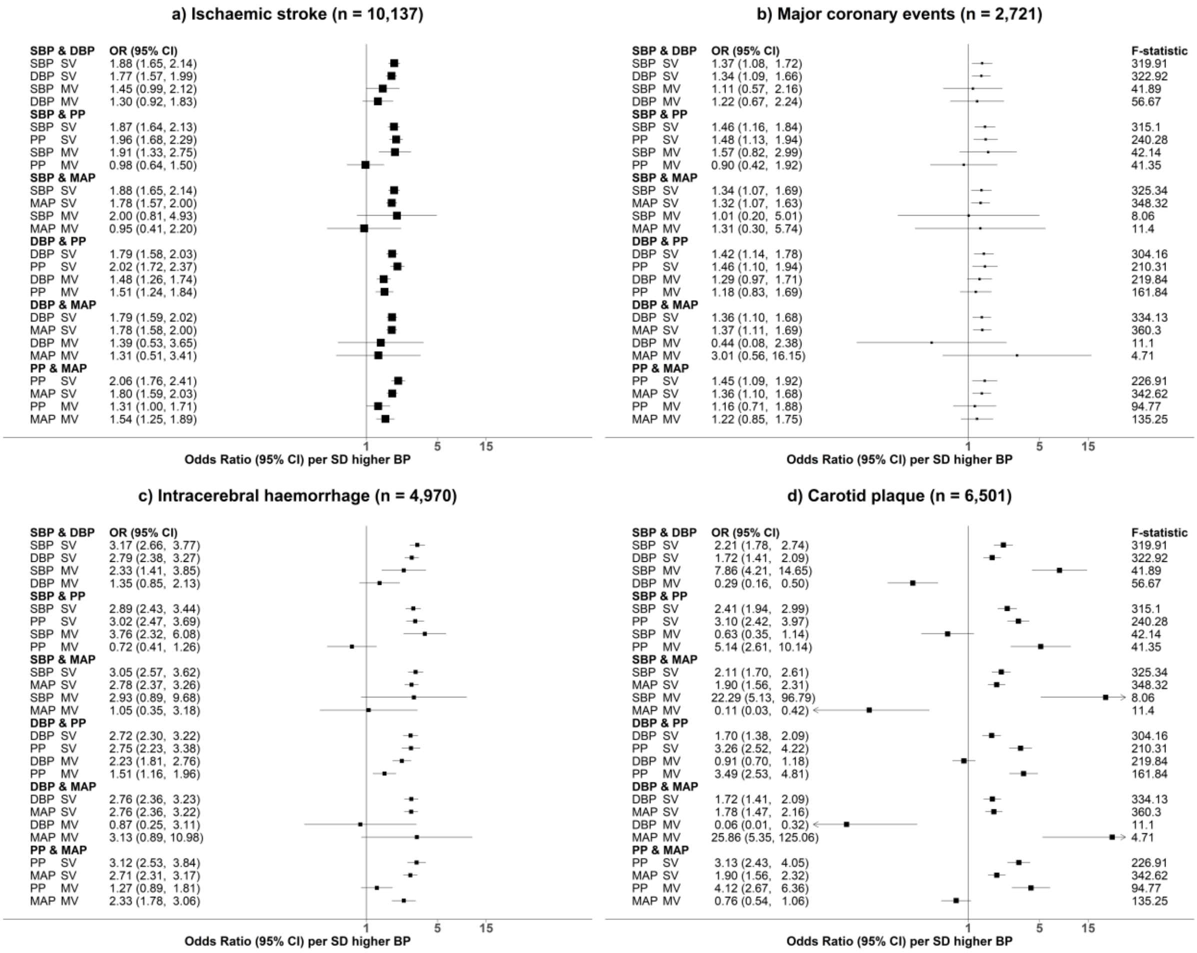

Supplementary Figure 17. Multivariable Mendelian randomisation of blood pressure with major cardiovascular diseases and subclinical atherosclerosis, sensitivity analysis. Effects are shown as odds ratios (95% CI) of disease risk per 1 SD higher BP trait. Loci from two traits were merged and in cases where more than one variant was available in the locus, the variant from the second trait was always included as the instrument when constructing the polygenic score. SBP indicates systolic blood pressure; DBP, diastolic blood pressure; PP, pulse pressure; MAP, mean arterial pressure; SV, single variable MR; MV, multi-variable MR. F-statistic was calculated as the average across CKB regions. F-statistic for MV indicates conditional F-statistic.

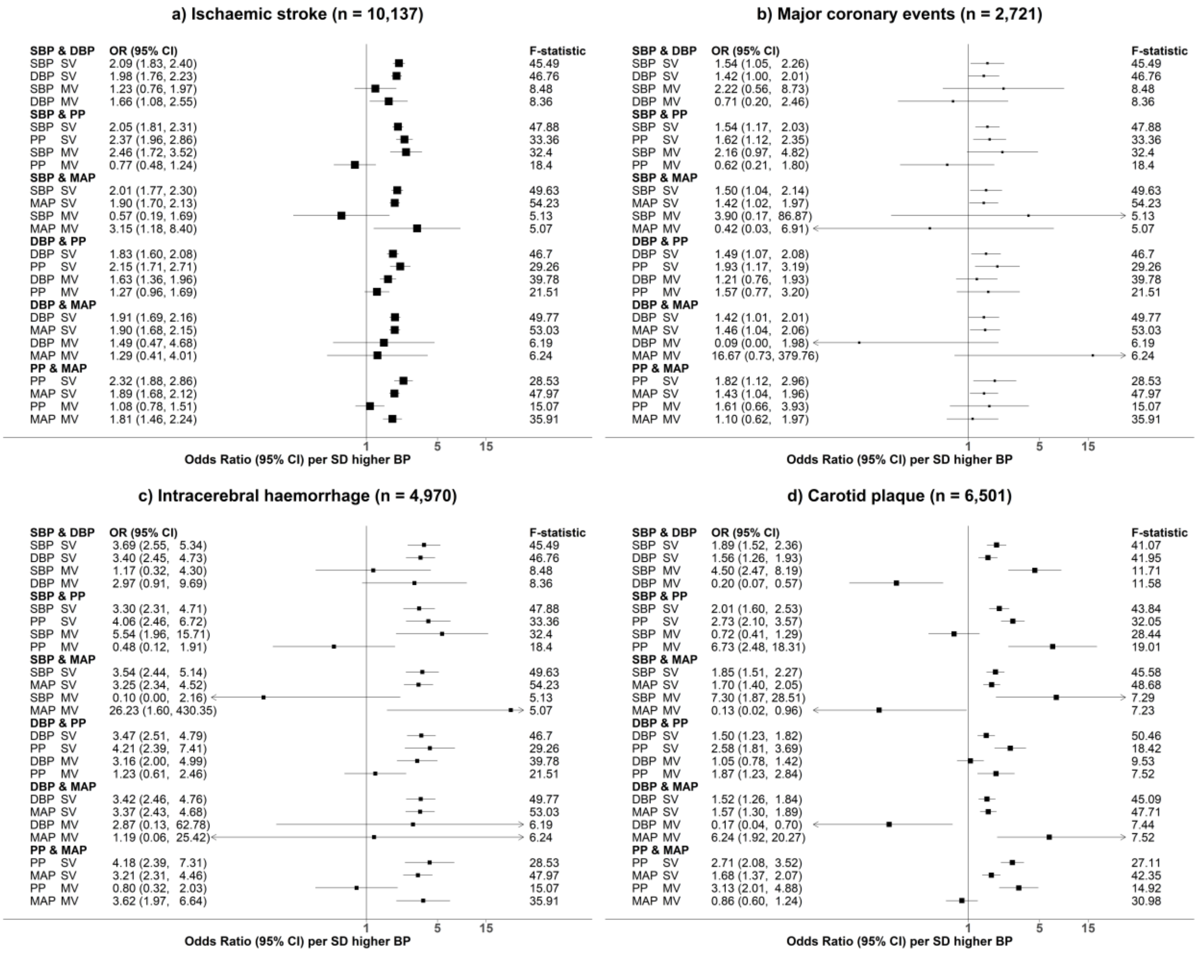

Supplementary Figure 18. Two-sample multivariable Mendelian randomisation of CKB blood pressure exposures with major cardiovascular diseases in BBJ and subclinical atherosclerosis in CKB. Effects are shown as odds ratios (95% CI) of disease risk per 1 SD higher BP trait. For each pair of traits, overlapping associated loci were merged and, in cases where there was more than one lead variant at a locus, the variant with the lowest P-value for assocaition with either trait was selected for inclusion in the GS. Note that for carotid plaque (CP, panel d), BP effect sizes were re-estimated in CKB after excluding individuals used to estimate CP effect sizes. SBP, systolic blood pressure; DBP, diastolic blood pressure; PP, pulse pressure; MAP, mean arterial pressure; SV, single variable MR; MV, multi-variable MR. F-statistic for MV indicates conditional F-statistic.

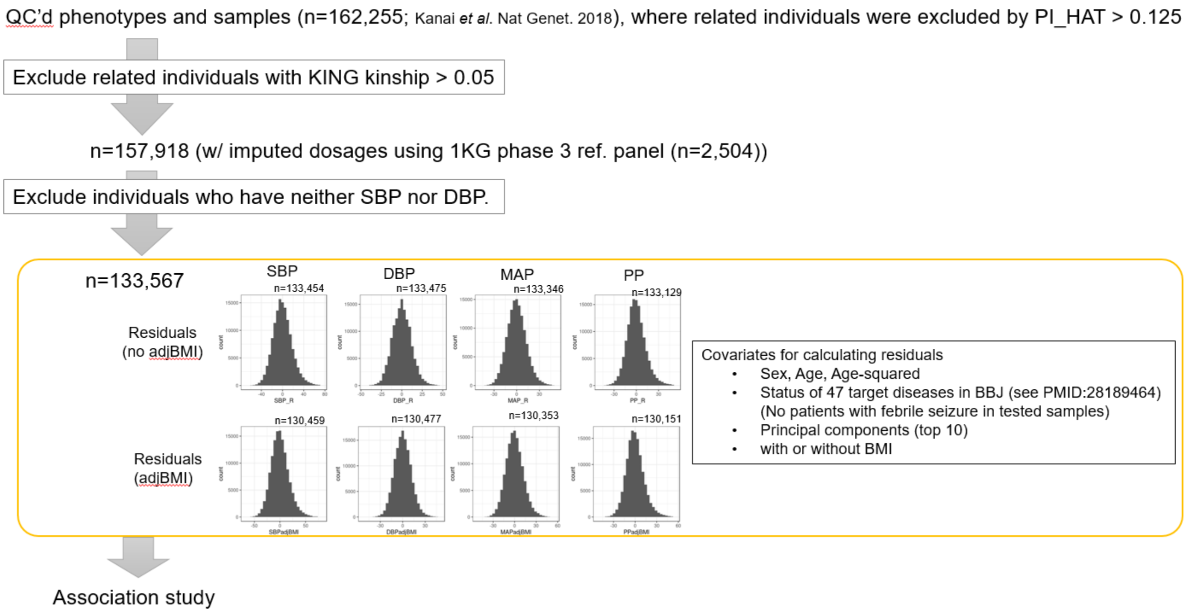

Supplementary Figure 19. Analysis workflow for replication of novel SNPs in BioBank Japan.
