# Supplementary material for: Causal relevance of different blood pressure traits on risk of cardiovascular diseases: GWAS and Mendelian randomisation in 100,000 Chinese adults
Source: Nat Commun. 2024 Jul 24;15:6265. doi: 10.1038/s41467-024-50297-x (PMC11269703; doi:10.1038/s41467-024-50297-x)
Supplement: Supplementary file 3 — Description of Additional Supplementary Files [file 41467_2024_50297_MOESM3_ESM.pdf]

Description of Additional Supplementary Files

File Name: Supplementary Data 1
Description: Baseline population characteristics of participants included in the China Kadoorie Biobank GWAS analyses

File Name: Supplementary Data 2
Description: Blood pressure population characteristics of participants

File Name: Supplementary Data 3
Description: Phenotypic correlations of BP traits in CKB

File Name: Supplementary Data 4
Description: Number of loci associated with blood pressure traits

File Name: Supplementary Data 5
Description: Genome-wide significant associations for BMI-unadjusted SBP

File Name: Supplementary Data 6
Description: Genome-wide significant associations for BMI-adjusted SBP

File Name: Supplementary Data 7
Description: Genome-wide significant associations for BMI-unadjusted DBP

File Name: Supplementary Data 8
Description: Genome-wide significant associations for BMI-adjusted DBP

File Name: Supplementary Data 9
Description: Genome-wide significant associations for BMI-unadjusted PP

File Name: Supplementary Data 10
Description: Genome-wide significant associations for BMI-adjusted PP

File Name: Supplementary Data 11
Description: Genome-wide significant associations for BMI-unadjusted MAP

File Name: Supplementary Data 12
Description: Genome-wide significant associations for BMI-adjusted MAP

File Name: Supplementary Data 13
Description: Results for the 18 additional secondary signals from conditional analysis

File Name: Supplementary Data 14
Description: Replication of novel associations in Biobank Japan and International Consortium of Blood Pressure

File Name: Supplementary Data 15
Description: BP trait heritabilities from meta-analysis of urban and rural regions of CKB

File Name: Supplementary Data 16
Description: Heritability in different strata estimated using REML

File Name: Supplementary Data 17
Description: Genetic correlation of blood pressure between Chinese (CKB) and Japanese (BBJ) populations

File Name: Supplementary Data 18
Description: Cross-ancestry SNP-based heritability and genetic correlation of blood pressure trait in Chinese (CKB) and European (ICBP), and in Japanese (BBJ) and European (ICBP) population

File Name: Supplementary Data 19
Description: Association of GS with BMI-adjusted blood pressure phenotypes in Mendelian randomisation

File Name: Supplementary Data 20
Description: Two-Sample Mendelian randomization between CKB BMI-adjusted blood pressure exposures and BBJ CVD outcomes (IS, ICH, MI) and CKB carotid plaque

File Name: Supplementary Data 21
Description: Association of GS with BMI-adjusted blood pressure phenotypes in multivariable Mendelian randomisation

File Name: Supplementary Data 22
Description: Two-sample multivariable MR between CKB BMI-adjusted blood pressure exposures and BBJ CVD outcomes (IS, ICH, MI) and CKB carotid plaque

File Name: Supplementary Data 23
Description: Estimates of heritability using BOLT-REML

File Name: Supplementary Data 24
Description: Assessment of genomic inflation using LD score regression
